# Guanidine acetic acid supplementation altered plasma and tissue free amino acid profiles in finishing pigs

**DOI:** 10.1186/s40813-022-00269-8

**Published:** 2022-06-07

**Authors:** Yiyan Cui, Zhimei Tian, Miao Yu, Dun Deng, Huijie Lu, Min Song, Xianyong Ma, Limin Wang

**Affiliations:** 1 Institute of Animal Science, Guangdong Academy of Agricultural Sciences, State Key Laboratory of Livestock and Poultry Breeding, The Key Laboratory of Animal Nutrition and Feed Science in South China, Ministry of Agriculture, Guangdong Provincial Key Laboratory of Animal Breeding and Nutrition, Guangdong Engineering Technology Research Center of Animal Meat Quality and Safety Control and Evaluation, Guangzhou, 510640 China; 2 Maoming Branch, Guangdong Laboratory for Lingnan Modern Agriculture, Maoming, 525000 China; 3 Guanghui Animal Husbandry Co., Ltd., Shaoguan, 512000 China

**Keywords:** Guanidine acetic acid, Pigs, Free amino acids

## Abstract

**Background:**

As a nutritive feed additive, guanidine acetic acid (GAA) participates in the metabolism of energy and proteins. This study aimed to investigate the effects of GAA on growth performance, organ index, plasma and tissue free amino acid profiles, and related metabolites in finishing pigs. A total of 72 crossbred pigs (body weight 86.59 ± 1.16 kg) were randomly assigned to 1 of 4 dietary treatments (GAA0, GAA500, GAA1000, and GAA1500). They were fed the basal diets supplemented with 0, 500, 1000, or 1500 mg/kg GAA for 42 days, respectively. The growth performance and organ weight were evaluated, and the contents of crude protein, free amino acids, and metabolites in plasma and tissues were determined. Spearman correlation between plasma and tissue free amino acids and related metabolites was also analyzed.

**Results:**

Growth performance in pigs was not altered by GAA (*P* > 0.05). The absolute and relative weight of kidneys increased (quadratic, *P* < 0.05). As dietary GAA concentration was increased, the contents of plasma glycine, serine, leucine, ornithine, and ratio of ornithine/arginine decreased (linear or quadratic, *P* < 0.05), but the contents of plasma isoleucine and taurine and the ratios of alanine/branched-chain amino acids and proline/ornithine increased quadratically (*P* < 0.05). The hepatic γ-amino-n-butyric acid content increased linearly and quadratically (*P* < 0.001), while the carnosine content decreased (quadratic, *P* = 0.004). The contents of renal arginine, proline, cystine, glutamate, and total amino acids (TAA) decreased quadratically (*P* < 0.05), but the contents of glycine (quadratic, *P* = 0.015) and γ-amino-n-butyric acid (linear, *P* = 0.008) increased. The pancreatic tryptophan content (quadratic, *P* = 0.024) increased, while the contents of pancreatic proline (linear, *P* = 0.005) and hydroxyproline (quadratic, *P* = 0.032) decreased in response to GAA supplementation. The contents of cardiac essential amino acids (EAA), nonessential amino acids (NEAA), and TAA in GAA1000 were higher than those in GAA1500 (*P* < 0.05). Supplementing with GAA linearly increased the contents of methionine, threonine, valine, isoleucine, leucine, phenylalanine, tryptophan, lysine, histidine, arginine, serine, alanine, glutamine, asparagine, tyrosine, proline, taurine, cystathionine, α-aminoadipic acid, β-aminoisobutyric acid, EAA, NEAA, and TAA in the spleen (*P* < 0.05). A strong Spearman correlation existed between plasma and tissue free amino acids and related metabolites.

**Conclusion:**

GAA supplementation did not altered pig growth performance, but it altered plasma and tissue free amino acid profiles and the contents of related metabolites in pigs in a tissue-dependent manner.

## Background

Guanidine acetic acid (GAA) is a metabolic intermediate, which is synthesized by arginine and glycine in the kidney, liver, and pancreas. It is then methylated to produce creatine in the liver [1–3], and then participates in the metabolism of energy and proteins [4]. As a nutritive feed additive, GAA has been approved by the European Food Safety Authority [5], the U. S. Food and Drug Administration [6], and the Ministry of Agriculture of China [7] for animals. Many previous studies have described the improvement in growth performance [8–10] and muscle performance [11–13] by GAA. Some of these studies focused on the addition of GAA under stress conditions [14, 15], the difference in supplementing GAA in an amino acid-deficient diet [16, 17], and the application of creatine replacement [18, 19]. Other studies focuses on improving high-intensity exercise performance [1, 20, 21]. However, data on the effect of GAA on tissue free amino acid profiles in finishing pigs and the correlation between tissue fand plasma free amino acids are limited. The visceral-bond amino acids reflect the composition of all amino acid units (proteins, polypeptides, etc.) in the tissues, mainly reflecting protein deposition. Free amino acids are generally used to reflect tissue amino acid metabolism, which serve as the cornerstone of protein synthesis, precursors of various biologically active molecules, and energy metabolites in tissues [22]. Systemic amino acid homeostasis depends on inter-organ metabolism. Amino acid contents in plasma and tissues reflect the dynamic balance between amino acid supply and use of amino acids in protein synthesis, gluconeogenesis, or catabolism [23, 24]. The liver and kidney play a key role in metabolism. Meanwhile, the kidney, liver, and pancreas are the main organs where GAA is produced, and the heart and spleen play a key role in systemic metabolism. In this study, we measured the growth performance, organ weight, organ protein level, plasma and tissue free amino acid profiles, and related metabolites in finishing pigs fed a GAA diet, focusing on the changes in plasma and tissue free amino acid profiles and the correlation among them.

## Results

### Growth performance

As shown in Table 1, growth performance in pigs was not altered by GAA (*P* > 0.05).

**Table 1:** Effects of guanidinoacetic acid (GAA) on growth performance in finishing pigs

Items	GAA, mg/kg				SEM	*P*-value		
	0	500	1000	1500		ANOVA	Linear	Quadratic
Initial weight, kg	86.91	86.50	86.44	86.50	3.99	1.000	0.943	0.954
Final weight, kg	127.5	126.51	131.28	125.25	4.44	0.799	0.922	0.579
Average daily gain, kg	0.97	0.95	1.07	0.92	0.05	0.238	0.937	0.223
Average daily feed intake, kg	3.16	3.39	3.52	3.15	0.16	0.289	0.877	0.070
Feed/gain	3.31	3.56	3.34	3.41	0.14	0.629	0.899	0.553

*SEM* standard error of the mean

Dietary treatments were: basal diet + 0, 500, 1000, and 1500 mg/kg GAA, respectively. n = 6

### Tissue absolute weight, relative weight, and crude protein

As shown in Table 2, the absolute weight (quadratic, *P* = 0.024) and relative weight (quadratic, *P* = 0.011) of kidneys increased as the dietary GAA concentration was increased. No significant difference was observed in the crude protein content of each tissue in each treatment (*P* > 0.05).

**Table 2:** Effects of guanidinoacetic acid (GAA) on absolute weight, relative weight, and crude protein of tissue in finishing pigs

Items	GAA, mg/kg				SEM	*P*-value		
	0	500	1000	1500		ANOVA	Linear	Quadratic
Weight, g								
Liver	1563.9	1662.7	1746.3	1637.4	99.1	0.678	0.525	0.335
Kidney	331.1	390.4	416.4	348.5	24.4	0.123	0.608	**0.024**
Pancreas	146.2	137.9	148.3	158.8	9.4	0.494	0.266	0.334
Heart	471.0	472.7	462.1	477.3	24.4	0.961	0.928	0.745
Spleen	218.5	194.2	208.9	201.0	14.3	0.690	0.577	0.588
Relative weight, g/kg body weight								
Liver	12.15	12.73	14.15	12.56	0.74	0.335	0.464	0.186
Kidney	2.55^b^	2.99^ab^	3.39^a^	2.67^ab^	0.19	**0.044**	0.509	**0.011**
Pancreas	1.13	1.06	1.22	1.22	0.08	0.440	0.253	0.660
Heart	5.09	5.05	4.91	5.00	0.20	0.934	0.668	0.759
Spleen	1.70	1.49	1.71	1.54	0.13	0.550	0.664	0.878
Crude protein, % dry tissue								
Liver	68.63	69.05	72.25	69.52	2.19	0.665	0.563	0.490
Kidney	73.12	73.44	75.09	71.79	1.01	0.296	0.758	0.141
Pancreas	62.85	61.37	68.64	63.03	3.65	0.554	0.649	0.590
Heart	71.00	71.51	70.99	70.32	1.09	0.901	0.614	0.602
Spleen	70.21	70.42	70.03	69.25	1.15	0.901	0.538	0.675

*SEM* standard error of the mean

Dietary treatments were: basal diet + 0, 500, 1000, and 1500 mg/kg GAA, respectively. n = 6

^a, b^Mean within rows with different letters differ significantly (*P* < 0.05)

Bold indicates *P* < 0.05

### Plasma free amino acid profile

As shown in Table 3, the contents of plasma glycine (linear, *P* = 0.002), serine (linear, *P* = 0.046), leucine (linear, *P* = 0.008), and ornithine (quadratic, *P* = 0.016) decreased as dietary GAA concentration was increased. The content of plasma isoleucine (quadratic, *P* = 0.035) and taurine (quadratic, *P* = 0.025) increased. The plasma TAA content in GAA1500 significantly decreased compared with that in GAA1000 (*P* = 0.036).

**Table 3:** Effects of guanidinoacetic acid (GAA) on plasma free amino acid profile in finishing pigs (nmol/mL)

Items	GAA, mg/kg				SEM	*P*-value		
	0	500	1000	1500		ANOVA	Linear	Quadratic
*EAA*								
Methionine	35.50	39.00	34.83	31.60	3.13	0.476	0.310	0.300
Threonine	128.33	141.00	138.67	114.60	9.56	0.286	0.413	0.083
Valine	224.67	243.33	236.83	191.00	15.24	0.132	0.179	0.051
Isoleucine	77.83	87.33	87.50	71.60	5.29	0.170	0.567	**0.035**
Leucine	195.67^ab^	205.67^a^	184.50^ab^	164.60^b^	8.25	**0.017**	**0.008**	0.081
Phenylalanine	75.00	78.17	73.17	67.20	3.46	0.202	0.096	0.196
Tryptophan	62.17	52.83	61.67	39.60	6.91	0.137	0.100	0.398
Lysine	192.17	183.83	193.67	146.60	12.86	0.130	0.086	0.205
Histidine	89.00	83.17	88.33	78.60	3.68	0.293	0.202	0.663
Arginine	147.33	136.83	142.50	129.20	7.49	0.515	0.240	0.888
*NEAA*								
Serine	122.33	118.83	108.83	99.80	7.57	0.228	**0.046**	0.728
Glycine	914.33^a^	863.00^ab^	845.33^ab^	727.00^b^	35.63	**0.014**	**0.002**	0.367
Alanine	354.00	342.83	341.00	361.40	17.96	0.843	0.807	0.394
Glutamate	74.83	84.83	80.83	74.60	7.38	0.748	0.939	0.307
Glutamine	233.00	256.17	261.83	252.60	17.11	0.689	0.415	0.384
Aspartic acid	17.40	15.50	21.67	15.80	2.48	0.393	0.850	0.498
Asparagine	30.50	35.75	44.83	31.40	4.81	0.270	0.554	0.104
Tyrosine	84.50	83.17	92.50	86.80	4.98	0.596	0.506	0.700
Proline	239.67	215.33	226.33	211.00	12.34	0.392	0.203	0.704
*Non-protein amino acids and derivatives/ metabolites of amino acids*								
Ornithine	59.17^ab^	67.00^a^	61.17^ab^	50.40^b^	3.57	**0.033**	0.079	**0.016**
Taurine	86.17	97.00	103.67	81.80	5.89	0.120	0.971	**0.025**
Citrulline	74.00	74.17	74.17	69.00	5.03	0.884	0.561	0.620
Hydroxyproline	101.00	127.00	133.67	126.00	16.34	0.570	0.296	0.352
Cystathionine	5.50	9.00	8.20	7.25	0.92	0.130	0.174	0.067
α-aminoadipic acid	39.67	37.67	43.00	39.00	2.94	0.822	0.864	0.829
1-Methyl-L-histidine	12.40	15.00	15.80	11.00	1.84	0.309	0.704	0.078
Urea	4261.33	4247.67	4330.50	3845.40	432.94	0.869	0.589	0.597
∑EAA	1165.50	1198.33	1180.00	995.00	47.30	0.061	0.053	0.054
∑NEAA	2053.17	1988.00	2001.50	1844.60	61.50	0.165	0.051	0.483
TAA	3671.17^ab^	3674.5^ab^	3695.67^a^	3273.2^b^	103.51	**0.036**	**0.032**	0.058

*SEM* standard error of the mean, *EAA* essential amino acids, *NEAA* nonessential amino acids, *TAA *total amino acids

Dietary treatments were: basal diet + 0, 500, 1000, and 1500 mg/kg GAA, respectively. n = 6

^a, b^Mean within rows with different letters differ significantly (*P* < 0.05)

Bold indicates *P* < 0.05

### Plasma ratio of amino acids

As shown in Table 4, the ratios of alanine/branched-chain amino acids (BCAA) (quadratic, *P* = 0.012) and proline/ornithine (quadratic, *P* = 0.009) increased, but the ratio of ornithine/arginine decreased (quadratic, *P* = 0.019) in response to GAA supplementation.

**Table 4:** Ratio of plasma amino acids in finishing pigs

Items	GAA, mg/kg				SEM	*P* -value		
	0	500	1000	1500		ANOVA	Linear	Quadratic
∑EAA/∑NEAA	0.57	0.60	0.59	0.54	0.03	0.424	0.523	0.130
Ornithine/arginine	0.41^ab^	0.49^a^	0.43^ab^	0.39^b^	0.02	**0.045**	0.347	**0.019**
Arginine/citrulline	2.00	1.91	2.00	1.90	0.16	0.952	0.789	0.996
Citrulline/ornithine	1.30	1.10	1.21	1.37	0.09	0.194	0.505	0.052
Proline/ornithine	4.19	3.23	3.71	4.18	0.24	0.062	0.794	**0.009**
Tyrosine/Phenylalanine	1.13	1.08	1.27	1.31	0.08	0.165	0.058	0.602
Valine/glycine	0.25	0.29	0.28	0.26	0.02	0.660	0.700	0.245
Serine/glycine	0.14	0.14	0.13	0.14	0.01	0.928	0.894	0.797
Alanine/BCAA	0.72^ab^	0.64^b^	0.68^ab^	0.86^a^	0.05	**0.028**	0.074	**0.012**

*GAA* guanidinoacetic acid, *SEM *standard error of the mean, *EAA* essential amino acids, *NEAA* nonessential amino acids, *BCAA *branched chain amino acids

Dietary treatments were: basal diet + 0, 500, 1000, and 1500 mg/kg GAA, respectively. n = 6

^a, b^Mean within rows with different letters differ significantly (*P* < 0.05)

Bold indicates *P* < 0.05

### Hepatic free amino acid profile

As shown in Table 5, the γ-amino-n-butyric acid content increased linearly and quadratically in the liver (*P* < 0.001), which was higher in the pigs fed a GAA diet than in the non-addition treatment (*P* < 0.001). The carnosine content decreased (quadratic, *P* = 0.004) as dietary GAA concentration was increased.

**Table 5:** Effects of guanidinoacetic acid (GAA) on hepatic free amino acid profile in finishing pigs (mg/kg dry liver)

Items	GAA, mg/kg				SEM	*P*-value		
	0	500	1000	1500		ANOVA	Linear	Quadratic
*EAA*								
Threonine	2.43	2.17	2.47	2.21	0.15	0.427	0.603	0.990
Valine	3.06	2.71	3.02	2.72	0.16	0.301	0.357	0.903
Isoleucine	1.05	0.93	1.13	1.02	0.07	0.323	0.701	0.928
Leucine	7.24	6.17	7.29	6.40	0.41	0.169	0.468	0.835
Phenylalanine	2.88	2.40	2.93	2.63	0.18	0.163	0.779	0.626
Tryptophan	3.31	3.50	3.38	3.65	0.71	0.987	0.775	0.955
Lysine	3.27	2.77	3.42	3.03	0.24	0.309	0.946	0.831
Histidine	2.36	2.04	2.20	2.13	0.17	0.596	0.489	0.441
Arginine	0.67	0.51	0.78	0.66	0.07	0.146	0.502	0.840
*NEAA*								
Serine	3.25	3.05	3.88	2.91	0.38	0.327	0.908	0.330
Glycine	9.93	10.02	10.15	8.81	0.46	0.183	0.133	0.140
Alanine	10.24	8.48	8.59	8.81	1.63	0.871	0.586	0.564
Glutamate	12.82	10.00	12.24	9.99	1.28	0.328	0.311	0.834
Glutamine	3.12	2.68	2.70	2.53	0.57	0.900	0.503	0.818
aspartic acid	3.00	2.12	3.37	2.49	0.41	0.227	0.895	0.991
Asparagine	1.26	1.23	1.32	1.29	0.09	0.925	0.688	0.950
Cystine	0.07	0.05	0.06	0.06	0.01	0.673	0.743	0.494
Tyrosine	2.41	1.98	2.41	2.18	0.17	0.306	0.756	0.611
Proline	6.39	6.30	6.35	7.01	0.80	0.939	0.644	0.687
*Non-protein amino acids and derivatives/metabolites of amino acids*								
Ornithine	1.81	1.72	1.93	2.04	0.16	0.614	0.273	0.577
Taurine	23.79	25.21	24.74	22.87	2.19	0.883	0.749	0.469
Citrulline	0.08	0.08	0.09	0.08	0.01	0.902	0.873	0.590
β-alanine	0.62	0.52	0.55	0.69	0.08	0.472	0.504	0.159
Cystathionine	3.39	2.83	3.36	3.08	0.23	0.300	0.699	0.554
α-aminoadipic acid	2.36	2.62	2.72	2.26	0.47	0.892	0.934	0.459
α-amino-n-butyric acid	1.11	0.78	1.13	1.10	0.13	0.221	0.595	0.287
β-aminoisobutyric acid	0.88	0.71	0.86	0.75	0.09	0.437	0.576	0.724
γ-amino-n-butyric acid	0.05^c^	0.30^b^	0.34^b^	0.41^a^	0.01	** < 0.001**	** < 0.001**	** < 0.001**
Anserine	1.93	1.26	2.03	1.50	0.20	0.069	0.668	0.789
Carnosine	1.68^a^	1.29^ab^	0.90^b^	1.78^a^	0.19	**0.014**	0.936	**0.004**
Ethanolamine	0.84	0.89	0.86	0.93	0.18	0.987	0.775	0.955
Urea	3.89	5.79	3.94	4.44	0.81	0.309	0.996	0.178
Ammonia	1.63	1.54	1.55	1.57	0.06	0.753	0.549	0.394
∑EAA	26.26	23.19	26.27	24.45	1.47	0.408	0.727	0.679
∑NEAA	86.53	80.57	86.59	79.20	4.46	0.566	0.454	0.880
TAA	112.75	103.69	112.73	103.50	5.85	0.530	0.498	0.989

*SEM* standard error of the mean, *EAA* essential amino acids, *NEAA* nonessential amino acids, *TAA* total amino acids

Dietary treatments were: basal diet + 0, 500, 1000, and 1500 mg/kg GAA, respectively. n = 6

^a, b, c^ Mean within rows with different letters differ significantly (*P* < 0.05)

Bold indicates *P* < 0.05

### Renal free amino acid profile

As shown in Table 6, the contents of arginine, proline, cystine, glutamate, NEAA, and TAA (linear or quadratic, *P* < 0.05) in the kidney decreased as dietary GAA concentration was increased. However, the contents of glycine (quadratic, *P* = 0.015) and γ-amino-n-butyric acid (linear, *P* = 0.008) increased.

**Table 6:** Effects of guanidinoacetic acid (GAA) on renal free amino acid profile in finishing pigs (mg/kg dry kidney)

Items	GAA, mg/kg				SEM	*P*-value		
	0	500	1000	1500		ANOVA	Linear	Quadratic
*EAA*								
Methionine	0.13	0.17	0.16	0.13	0.02	0.278	0.700	0.063
Threonine	7.15	8.36	8.25	7.54	0.61	0.504	0.717	0.151
Valine	8.36	9.73	9.19	8.68	0.74	0.639	0.909	0.249
Isoleucine	4.21	4.60	4.54	3.94	0.36	0.594	0.621	0.208
Leucine	16.29	17.78	17.25	15.63	1.31	0.705	0.692	0.280
Phenylalanine	7.61	8.79	8.30	7.03	0.65	0.317	0.479	0.091
Tryptophan	11.57	12.18	11.68	11.83	1.10	0.983	0.957	0.845
Lysine	9.24	11.16	10.61	9.09	0.93	0.364	0.820	0.089
Histidine	3.20	3.98	3.91	3.87	0.30	0.332	0.203	0.229
Arginine	6.27	7.89	7.04	5.74	0.64	0.158	0.430	**0.043**
*NEAA*								
Serine	10.53	12.28	12.22	10.54	1.04	0.503	0.993	0.135
Glycine	24.22	30.83	32.64	26.40	2.37	0.080	0.446	**0.015**
Alanine	17.67	19.34	19.89	17.81	1.35	0.646	0.883	0.219
Glutamate	37.40^b^	49.99^a^	47.15^ab^	37.96^b^	2.59	**0.010**	0.930	**0.001**
Glutamine	0.51	0.81	0.77	0.52	0.13	0.270	0.997	0.055
Aspartic acid	10.46	11.91	11.50	10.82	0.91	0.709	0.879	0.279
Asparagine	3.78	4.59	5.04	4.64	0.42	0.263	0.139	0.188
Cystine	3.21^b^	9.00^a^	6.69^ab^	3.02^b^	1.35	**0.017**	0.646	**0.003**
Tyrosine	6.96	8.17	7.51	6.57	0.60	0.329	0.526	0.103
Proline	19.25^a^	22.25^a^	18.07^a^	8.68^b^	1.96	**0.002**	**0.001**	**0.009**
*Non-protein amino acids and derivatives/metabolites of amino acids*								
Ornithine	1.34	1.77	1.85	1.68	0.16	0.173	0.150	0.087
Taurine	31.65	34.05	32.50	35.24	2.04	0.673	0.364	0.941
Citrulline	1.02	1.01	1.04	1.00	0.03	0.900	0.827	0.622
β-alanine	1.69	1.71	1.49	1.54	0.10	0.550	0.251	0.895
Hydroxyproline	1.18	1.49	1.10	1.35	0.18	0.543	0.869	0.946
Cystathionine	7.79	8.86	8.39	7.50	0.67	0.517	0.670	0.170
α-aminoadipic acid	5.44	3.75	4.81	8.62	1.15	0.089	0.089	0.051
γ-amino-n-butyric acid	0.36^b^	0.46^ab^	0.45^ab^	0.53^a^	0.04	**0.039**	**0.008**	0.793
Urea	26.94	32.75	19.66	27.93	6.21	0.561	0.729	0.851
Ammonia	1.84^a^	1.93^a^	1.58^ab^	0.96^b^	0.15	**0.001**	**0.000**	**0.036**
∑EAA	74.01	84.58	80.92	73.42	6.04	0.558	0.852	0.177
∑NEAA	184.98	222.48	213.45	184.42	12.15	0.131	0.861	**0.023**
TAA	258.13	306.04	293.71	256.84	18.05	0.222	0.856	**0.045**

*SEM* standard error of the mean, *EAA* essential amino acids, *NEAA* nonessential amino acids, *TAA* total amino acids

Dietary treatments were: basal diet + 0, 500, 1000, and 1500 mg/kg GAA, respectively. n = 6

^a, b^Mean within rows with different letters differ significantly (*P* < 0.05)

Bold indicates *P* < 0.05

### Pancreatic free amino acid profile

As shown in Table 7, the pancreatic tryptophan content increased as the dietary GAA concentration was increased (quadratic, *P* = 0.024), but the tryptophan content of GAA1500 was lowest (*P* = 0.042). The contents of pancreatic proline (linear, *P* = 0.005) and hydroxyproline (quadratic, *P* = 0.032) decreased. Compared with GAA0 and GAA500, the contents of ethanolamine and urea in GAA1500 increased (*P* < 0.05).

**Table 7:** Effects of guanidinoacetic acid (GAA) on pancreatic free amino acid profile in finishing pigs (mg/kg dry pancreas)

Items	GAA, mg/kg				SEM	*P*-value		
	0	500	1000	1500		ANOVA	Linear	Quadratic
*EAA*								
Methionine	0.40	0.35	0.45	0.27	0.09	0.538	0.464	0.429
Threonine	2.86	1.77	2.08	1.56	0.33	0.113	**0.047**	0.456
Valine	3.83	2.63	3.05	2.12	0.44	0.136	**0.049**	0.792
Isoleucine	1.52	1.15	1.43	0.85	0.20	0.161	0.090	0.629
Leucine	9.45	7.20	8.46	5.87	1.21	0.259	0.116	0.894
Phenylalanine	5.33	4.10	4.97	3.21	0.63	0.125	0.072	0.685
Tryptophan	1.68^a^	1.77^a^	2.15^a^	0.62^b^	0.33	**0.042**	0.128	**0.024**
Lysine	5.37	4.09	5.04	3.24	0.75	0.251	0.140	0.743
Histidine	1.27	0.94	1.32	1.27	0.20	0.900	0.854	0.737
Arginine	3.58	2.86	3.47	2.50	0.66	0.644	0.399	0.859
*NEAA*								
Serine	5.29	4.31	4.97	4.09	0.63	0.534	0.321	0.938
Glycine	10.27	9.64	10.96	8.67	1.09	0.515	0.486	0.457
Alanine	12.02	11.36	11.10	11.93	1.19	0.937	0.924	0.543
Glutamate	18.67	19.58	20.25	17.45	2.12	0.865	0.790	0.459
Glutamine	0.35	0.27	0.47	0.47	0.11	0.442	0.237	0.663
Aspartic acid	5.31	4.41	4.52	3.62	0.66	0.405	0.121	0.996
Asparagine	2.52	1.37	1.66	1.30	0.31	0.114	0.057	0.310
Cystine	0.37	0.34	0.43	0.29	0.04	0.423	0.658	0.273
Tyrosine	4.76	4.00	4.51	3.47	1.02	0.825	0.480	0.891
Proline	4.00^a^	3.42^ab^	3.13^ab^	2.48^b^	0.33	**0.036**	**0.005**	0.930
*Non-protein amino acids and derivatives/metabolites of amino acids*								
Ornithine	1.37	1.31	1.38	1.17	0.15	0.830	0.537	0.654
Taurine	6.57	6.31	6.28	6.70	0.70	0.974	0.914	0.658
Hydroxyproline	1.18	1.32	1.29	0.89	0.11	0.051	0.067	**0.032**
Cystathionine	3.72	2.61	3.16	2.22	0.45	0.173	0.082	0.860
a-aminoadipic acid	1.32	1.21	1.38	1.47	0.22	0.870	0.549	0.658
a-amino-n-butyric acid	0.39	0.27	0.39	0.28	0.08	0.556	0.573	0.782
Phosphoethanolamine	16.54	16.99	15.47	17.21	2.02	0.936	0.957	0.761
Ethanolamine	0.50^b^	0.43^b^	0.37^b^	2.28^a^	2.02	**0.001**	**0.001**	**0.002**
Urea	0.42^b^	0.39^b^	0.59^ab^	1.07^a^	0.21	**0.015**	**0.004**	0.123
∑EAA	33.60	25.14	31.32	20.87	4.46	0.247	0.148	0.836
∑NEAA	77.45	71.58	75.84	66.40	7.10	0.711	0.384	0.808
TAA	111.04	96.72	107.16	87.27	11.15	0.473	0.250	0.811

*SEM* standard error of the mean, *EAA* essential amino acids, *NEAA* nonessential amino acids, *TAA* total amino acids

Dietary treatments were: basal diet + 0, 500, 1000, and 1500 mg/kg GAA, respectively. n = 6

^a, b^Mean within rows with different letters differ significantly (*P* < 0.05)

Bold indicates *P* < 0.05

### Cardiac free amino acid profile

As shown in Table 8, the contents of cardiac serine (quadratic, *P* = 0.009), glycine (quadratic, *P* = 0.008), and ammonia (linear, *P* < 0.001) increased with the increase in the GAA content, but the glutamine content decreased linearly (*P* = 0.038). The contents of ornithine (vs GAA0, GAA500, and GAA1500) and β-aminoisobutyric acid (vs GAA500 and GAA1500) in GAA1000 increased significantly (*P* < 0.05). The contents of cardiac EAA, NEAA, and TAA were higher in GAA1000 than those in GAA1500 (*P* < 0.05).

**Table 8:** Effects of guanidinoacetic acid (GAA) on cardiac free amino acid profile in finishing pigs (mg/kg dry heart)

Items	GAA, mg/kg				SEM	*P* -value		
	0	500	1000	1500		ANOVA	Linear	Quadratic
*EAA*								
Threonine	0.85	1.02	1.00	0.67	0.15	0.436	0.455	0.150
Valine	0.74	0.84	0.91	0.76	0.08	0.412	0.774	0.121
Isoleucine	0.31	0.31	0.33	0.26	0.02	0.083	0.137	0.058
Leucine	1.46	1.42	1.69	1.57	0.10	0.248	0.189	0.722
Phenylalanine	0.74	0.66	0.81	0.74	0.04	0.113	0.447	0.783
Tryptophan	0.11	0.09	0.11	0.12	0.02	0.625	0.406	0.354
Lysine	1.39	1.41	1.65	1.27	0.10	0.112	0.776	0.073
Histidine	1.35	1.19	1.53	1.22	0.10	0.105	0.906	0.474
Arginine	1.20	1.13	1.27	1.05	0.06	0.129	0.313	0.242
*NEAA*								
Serine	0.87^b^	1.16^ab^	1.34^a^	0.97^ab^	0.11	**0.039**	0.342	**0.009**
Glycine	1.84^ab^	1.99^ab^	2.23^a^	1.53^b^	0.14	**0.020**	0.291	**0.008**
Alanine	16.62	16.99	18.79	16.55	0.85	0.294	0.706	0.168
Glutamate	4.88	5.08	5.66	4.95	0.55	0.763	0.758	0.433
Glutamine	61.14	62.10	59.66	48.53	3.90	0.097	**0.038**	0.152
aspartic acid	0.53	0.39	0.43	0.32	0.11	0.659	0.279	0.881
Asparagine	0.38	0.43	0.57	0.44	0.05	0.162	0.255	0.125
Tyrosine	0.71	0.68	0.68	0.64	0.04	0.561	0.185	0.889
Proline	0.90	1.07	0.97	1.03	0.10	0.659	0.547	0.584
*Non-protein amino acids and derivatives/ metabolites of amino acids*								
Ornithine	0.13^b^	0.13^b^	0.20^a^	0.13^b^	0.02	**0.025**	0.347	0.054
Taurine	47.93	42.31	54.58	43.34	3.89	0.167	0.935	0.500
Citrulline	0.71	0.90	1.21	0.97	0.24	0.598	0.338	0.440
Cystathionine	1.00	1.00	1.16	1.06	0.07	0.429	0.344	0.482
α-aminoadipic acid	0.45	0.33	0.41	0.44	0.06	0.580	0.866	0.246
β-aminoisobutyric acid	0.42^ab^	0.39^b^	0.57^a^	0.38^b^	0.04	**0.011**	0.885	**0.046**
Carnosine	1.49	0.97	0.86	1.32	0.19	0.228	0.577	0.050
Ethanolamine	0.40	0.32	0.42	0.44	0.05	0.578	0.480	0.356
Urea	3.29	4.47	2.88	3.41	0.52	0.237	0.620	0.564
Ammonia	2.09^b^	2.13^b^	2.42^ab^	2.89^a^	0.12	**0.001**	** < 0.001**	0.093
∑EAA	8.10^ab^	8.04^ab^	9.19^a^	7.61^b^	0.38	**0.046**	0.851	0.058
∑NEAA	138.43^ab^	134.87^ab^	147.57^a^	120.79^b^	4.71	**0.032**	0.142	0.062
TAA	146.53^ab^	142.91^ab^	156.76^a^	128.40^b^	5.71	**0.029**	0.152	0.056

*SEM* standard error of the mean, *EAA* essential amino acids, *NEAA* nonessential amino acids, *TAA* total amino acids

Dietary treatments were: basal diet + 0, 500, 1000, and 1500 mg/kg GAA, respectively. n = 6

^a, b^Mean within rows with different letters differ significantly (*P* < 0.05)

Bold indicates *P* < 0.05

### Splenic free amino acid profile

As shown in Table 9, supplementing with GAA increased the contents of methionine, threonine, valine, isoleucine, leucine, phenylalanine, tryptophan, lysine, histidine, arginine, serine, alanine, glutamine, asparagine, tyrosine, proline, taurine, cystathionine, α-aminoadipic acid, β-aminoisobutyric acid, EAA, NEAA, and TAA in the spleen (linear, *P* < 0.05). The contents of glycine (vs GAA0 and GAA500), aspartic acid (vs GAA0 and GAA500), glutamate (vs GAA0, GAA500, and GAA1500) increased significantly in GAA1000 (*P* < 0.01).

**Table 9:** Effects of guanidinoacetic acid (GAA) on splenic free amino acid profile in finishing pigs (mg/kg dry spleen)

Items	GAA, mg/kg				SEM	*P*-value		
	0	500	1000	1500		ANOVA	Linear	Quadratic
*EAA*								
Methionine	0.09	0.07	0.20	0.19	0.01	**0.002**	**0.001**	0.491
Threonine	3.28^b^	3.40^b^	5.17^a^	4.82^a^	0.16	** < 0.001**	** < 0.001**	0.159
Valine	4.70^b^	4.63^b^	7.31^a^	7.14^a^	0.18	** < 0.001**	** < 0.001**	0.695
Isoleucine	1.56^b^	1.57^b^	2.42^a^	2.32^a^	0.06	** < 0.001**	** < 0.001**	0.398
Leucine	12.16^b^	11.77^b^	18.51^a^	18.96^a^	0.54	** < 0.001**	** < 0.001**	0.538
Phenylalanine	4.48^b^	4.39^b^	6.82^a^	6.76^a^	0.19	** < 0.001**	** < 0.001**	0.965
Tryptophan	1.82^b^	1.83^b^	9.26^a^	9.10^a^	0.36	** < 0.001**	** < 0.001**	0.706
Lysine	5.24^b^	5.26^b^	7.82^a^	7.11^a^	0.29	** < 0.001**	** < 0.001**	0.208
Histidine	1.96^c^	1.90^c^	2.93^a^	2.57^b^	0.09	** < 0.001**	** < 0.001**	0.087
Arginine	4.14^b^	3.98^b^	6.29^a^	5.63^a^	0.21	** < 0.001**	** < 0.001**	0.287
*NEAA*								
Serine	4.41^b^	4.58^b^	7.05^a^	6.77^a^	0.21	** < 0.001**	** < 0.001**	0.312
Glycine	14.67^b^	14.54^b^	20.31^a^	17.07^ab^	0.97	**0.007**	**0.025**	0.179
Alanine	6.29^b^	6.54^b^	9.04^a^	9.36^a^	0.35	** < 0.001**	** < 0.001**	0.987
Glutamate	35.20^b^	33.73^b^	44.92^a^	36.18^b^	1.83	**0.004**	0.148	0.079
Glutamine	0.48^b^	0.43^b^	1.06^a^	1.06^a^	0.05	** < 0.001**	** < 0.001**	0.700
Aspartic acid	10.45^bc^	9.40^c^	15.11^a^	12.54^ab^	0.73	** < 0.001**	**0.002**	0.270
Asparagine	1.90^b^	1.93^b^	2.92^a^	2.56^a^	0.12	** < 0.001**	** < 0.001**	0.127
Cystine	0.19	0.24	0.23	0.23	0.04	0.895	0.691	0.597
Tyrosine	3.40^b^	3.39^b^	5.08^a^	5.06^a^	0.16	** < 0.001**	** < 0.001**	0.906
Proline	3.05^b^	2.71^b^	5.24^a^	5.20^a^	0.22	** < 0.001**	** < 0.001**	0.639
*Non-protein amino acids and derivatives/metabolites of amino acids*								
Ornithine	0.08	0.09	0.08	0.13	0.04	0.901	0.530	0.774
Taurine	31.59	31.81	33.36	37.29	1.77	0.171	**0.044**	0.352
Citrulline	0.27	0.26	0.28	0.35	0.03	0.250	0.085	0.295
β-alanine	0.49	0.35	0.44	0.42	0.06	0.571	0.771	0.366
Cystathionine	5.10^b^	5.01^b^	7.55^a^	8.15^a^	0.25	** < 0.001**	** < 0.001**	0.245
α-aminoadipic acid	1.70^b^	1.80^ab^	2.05^ab^	2.24^a^	0.11	**0.017**	**0.002**	0.718
α-amino-n-butyric acid	0.81	0.81	1.12	0.84	0.14	0.403	0.592	0.351
β-aminoisobutyric acid	1.23^b^	1.23^b^	1.42^ab^	1.68^a^	0.09	**0.012**	**0.002**	0.199
Phosphoethanolamine	11.92^ab^	9.92^b^	17.43^a^	14.03^ab^	1.38	**0.009**	**0.044**	0.584
Urea	2.47	3.20	2.60	3.66	0.32	0.347	0.197	0.956
Ammonia	2.45	2.08	2.18	1.97	0.15	0.200	0.076	0.602
∑EAA	39.37^b^	38.74^b^	66.66^a^	64.60^a^	1.49	** < 0.001**	** < 0.001**	0.549
∑NEAA	121.50^b^	119.18^b^	156.95^a^	146.85^a^	3.61	** < 0.001**	** < 0.001**	0.230
TAA	160.87^b^	157.92^b^	223.61^a^	211.45^a^	3.93	** < 0.001**	** < 0.001**	0.256

SEM standard error of the mean, EAA essential amino acids, NEAA nonessential amino acids, TAA total amino acids

Dietary treatments were: basal diet + 0, 500, 1000, and 1500 mg/kg GAA, respectively. n = 6

^a, b^Mean within rows with different letters differ significantly (*P* < 0.05)

Bold indicates *P* < 0.05

### Spearman correlation analysis between plasma and tissue free amino acids and related metabolites

**Fig.1:**
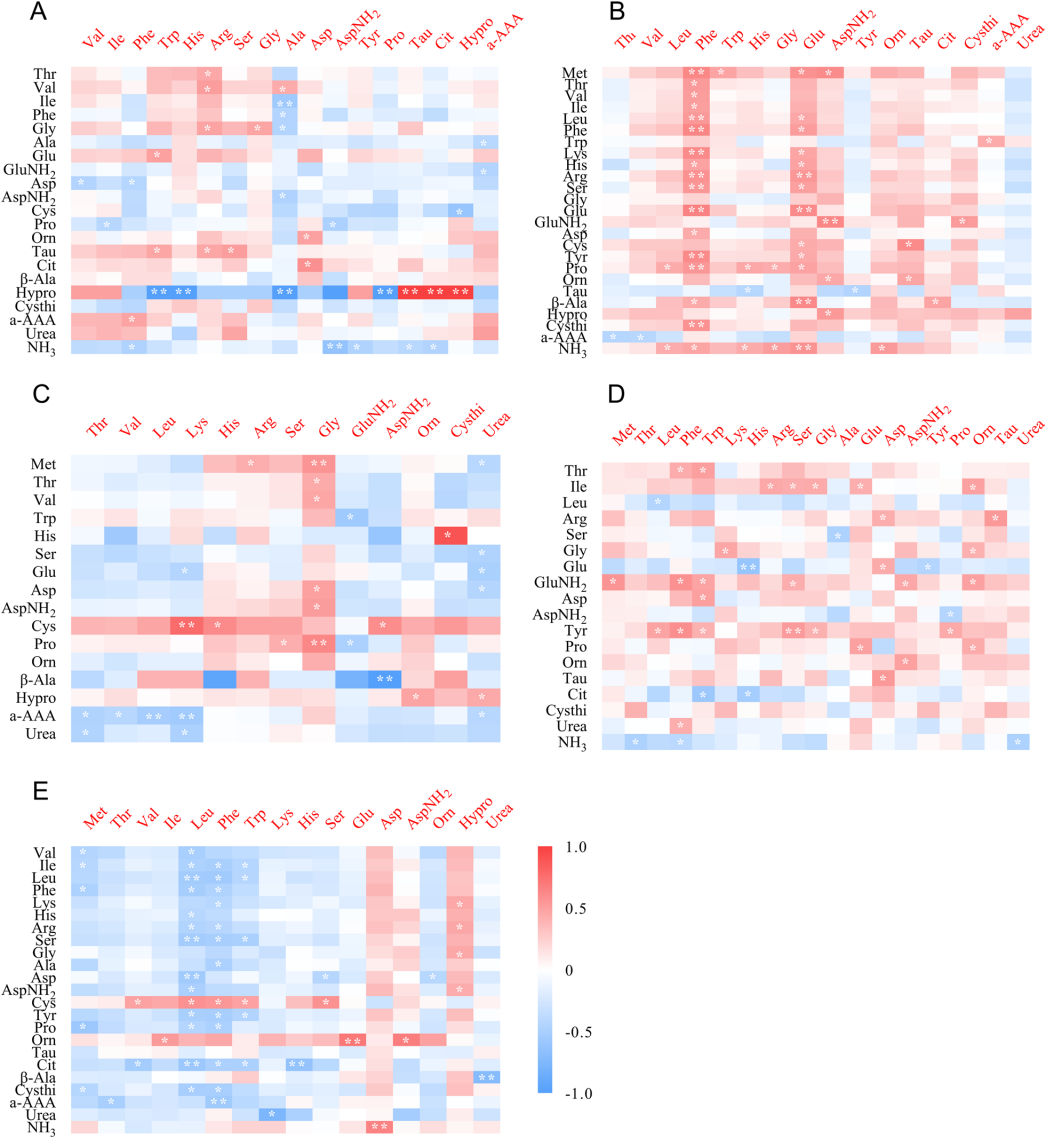
Spearman correlation analysis between plasma and tissue: **A** liver, **B** kidney, **C** pancreas, **D** heart, **E** spleen. Right colored bar indicates scale range of Spearman correlation coefficients depicted. Red denotes positive, whereas blue indicates negative. *P < 0.05; **P < 0.01. GluNH_2_ = glutamine; AspNH_2_ = asparagine; β-Ala = β-alanine; Hypro = hydroxyproline; Cysthi = cystathionine; a-AAA = a-aminoadipic acid; NH_3_ = ammonia

Spearman correlation analysis was conducted to explore the relationship between plasma and tissue free amino acids and related metabolites. A significant positive/negative strong correlation was found between hepatic hydroxyproline content and some amino acids in plasma (*ρ* = 1 or *ρ* = –1, *P* < 0.01; Fig. 1A). Kidney and plasma free amino acids were mainly positively correlated (Fig. 1B), but negatively between spleen and plasma (Fig. 1E). A certain correlation between the free amino acids in the pancreas, heart, and plasma was observed (Fig. 1C and Fig. 1D). These results indicated that these differential tissue amino acids were closely associated with, and might contribute to, the altered plasma amino acid profiles in response to GAA supplementation.

## Discussion

In this study, we found that GAA had no significant effect on the growth performance of finishing pigs. This result was consistent with previous findings, that is, dietary GAA (< 2000 mg/kg) did not affect the growth performance of pigs [11, 25, 26]. However, other studies reported that the addition of GAA improved the growth performance of finishing pigs [9, 10, 27]. The differences among these studies might be related to the gender, initial weight, treatment time, or nutritional value. In our study, no changes in plasma arginine/citrulline, EAA/NEAA, and valine/glycine ratios were found among the groups, indicating that the nutritional status of the pigs was consistent [28]. Pigs might have grown at their maximum speed and growth parameters did not decreased, resulting in no serious imbalance in plasma EAA.

The absolute and relative weight of organs can reflect the function of the body to a certain extent [29, 30]. Weight gain is accompanied by an increase in organ weight [31]. The absolute and relative weights of the liver, heart, spleen, and pancreas were not affected by GAA supplementation. The absolute and relative weight of the kidney increased as the dietary GAA concentration increased. The kidney is the main tissue for synthesizing GAA. The increase in the absolute and relative weight of kidneys might be related to the changes in energy metabolism, excretion function and protein deposition of kidney by GAA supplementation, and the specific mechanism needs further study.

Glycine is synthesized from serine, threonine, choline, and hydroxyproline through inter-organ metabolism in the liver and kidney [32]. The contents of plasma glycine and serine decreased linearly with the increase in the GAA content, while threonine content decreased quadratically. Glycine and serine could transform each other rapidly, and hence their changes were consistent [33]. Glycine is not only the precursor of GAA, but also participates in the synthesis of glutathione, purines, creatine, porphyrins of heme, and primary bile salts [34]. The decrease in the plasma glycine content might be due to the increase in its ability to synthesize other metabolites by GAA supplementation. However, the bovine plasma threonine content increased linearly with GAA supplementation [35]. Majdeddin et al. [15] reported that 0.6 g/kg GAA increased the plasma glycine and serine levels of broilers on day 39. However, DeGroot et al. [36] reported that 0.06–0.12% GAA did not affect the serum glycine content of broilers. This might be due to different animal species and time of addition.

In this study, the plasma ornithine content decreased and the citrulline/ornithine ratio increased with the increase in the GAA concentration. It indicated that ornithine catabolism was activated to promote citrulline and proline synthesis [28]. The proline/ornithine ratio increased with the increase in the GAA content, proving the aforementioned hypothesis. The results of this study were consistent with the findings of McBreairty et al. [26] and Liu et al. [37], showing that GAA supplementation caused no significant difference in the blood arginine content of pigs and cattle. However, Ardalan et al. [35] and Majdeddin et al. [15] reported that dietary GAA increased the plasma arginine concentration in heifers (limit-fed) and broilers (chronic circulatory heat stress). The aforementioned results were inconsistent, which might be due to the different effects of the heat stress model, diet restriction, and animal species on the synthesis and catabolism of amino acids.

The contents of plasma isoleucine, leucine, lysine, phenylalanine, valine, and threonine decreased with the increase in the dietary GAA concentration, which was consistent with the results of He et al. [38]. No significant difference was found in the plasma leucine content between GAA treatments in broilers [38]. Compared with GAA500, GAA1500 significantly reduced the plasma leucine content in this experiment. Leucine in all tissues (except the spleen) in the study was not affected by the GAA concentration. The change in the plasma leucine content might have originated from the spleen, intestines, or other tissues that we did not measured. The correlation analysis showed that the plasma leucine content negatively correlated with the splenic contents of valine, leucine, phenylalanine, serine, aspartic acid, tyrosine, citrulline, and cystathionine, but positively correlated with the splenic cystine content.

One study suggested that GAA supplementation reduced the serum amino acids concentration in broilers [36]. This was consistent with our findings. The plasma content of most amino acids decreased with the increase in the GAA concentration (linear or quadratic). GAA changed the pig amino acid metabolism. It could be directly methylated to creatine, which saved other amino acids for creatine synthesis and promoted their use for cell growth, division, signal transduction, etc. [39].

The catabolism of amino acids mainly occurs in the liver [40]. However, the amino acid profile of the liver was not significantly changed by GAA supplementation, which was consistent with the report by Ostojic et al. [41], showing that the hepatic enzyme profile was not affected by GAA. GAA acts as a possible activator of γ-aminobutyric acid receptors in the brain and peripheral tissues [39]. In this experiment, GAA increased γ-aminobutyric acid content in the liver and kidney, but γ-aminobutyric acid content in the brain was not measured. Whether exogenous GAA acts as a neuromodulator and affects the excitability and brain development is unclear. Muscle is the main storage site of carnosine [15]. β-alanine and histidine catalyze carnosine synthesis by adenosine triphosphate dependent carnosine synthase [21]. However, the contents of histidine and alanine in the liver and heart were unchanged. Therefore the changes in the carnosine content could not be explained by the synthesis of the aforementioned two amino acids.

The contents of methionine, phenylalanine, arginine, proline, cystine, glutamic acid, and glutamine in the kidney decreased with the increase in GAA content. Especially, compared with GAA500, GAA1500 significantly reduced the contents of glutamic acid, cystine, and glycine in the kidney. This indicated that the high GAA concentration increased renal gluconeogenesis, which increased the catabolism of the aforementioned glycogenic amino acids, decreasing the levels of these amino acids [42]. A previous study demonstrated the conversion of phenylalanine into tyrosine [43]. The renal tyrosine content was significantly positively correlated with the plasma phenylalanine content, and the trend of phenylalanine and tyrosine in the kidney was consistent, which might validate the aforementioned result. Renal excretion is accomplished by filtering the blood, reabsorbing salts, and circulating nutrients. A strong positive correlation was found between kidney and plasma amino acids. This might be due to renal reabsorption, where metabolites were reabsorbed more efficiently. Increased reabsorption efficiency avoided the wastage of large amounts of circulating metabolites [44].

Studies on the amino acid profiles of pancreas and heart were few. Why GAA changed some free amino acids in the pancreas and heart was unclear. In this experiment, no change was found in the absolute and relative weights of the pancreas and heart; also, no difference in pig growth performance was observed. It showed that GAA did not affect the pancreas and heart function of finishing pigs.

The spleen is the largest lymphoid organ, which can produce a large amount of antibodies [30]. Amino acids are the basic structural substances of the immune system [45]. The content of most amino acids increased linearly with the increase in GAA concentration. Most amino acids in the spleen were affected by GAA supplementation, which might change the immunity. The increase in the taurine level might be closely related to the degradation of splenic leukocytes [43, 46]. The changes in the taurine level in plasma and spleen were similar. The changes in amino acids in the spleen by GAA were opposite to those in plasma, liver, and kidney; splenic amino acids were mainly negatively correlated with plasma amino acids. GAA may change the immune status of the body by altering the splenic amino acid profile. The follow-up studies require the determination of immune indexes. A glutamatergic system exists in immune cells, and the increase in the glutamate content can stimulate lymphocyte activation and splenic immunity [47]. In addition, ammonia and glutamate can form glutamine, which is the main method of ammonia detoxification [48]. In this study, the glutamine contentwas significantly higher in GAA1000 and GAA1500 than in GAA0 and GAA500. The glutamate content was significantly higher in GAA1000 than in other groups. We speculated that the ammonia concentration in the spleen was higher in GAA than that in GAA0, which led to a high glutamine content in GAA groups. However, no significant difference was noted in the splenic ammonia content among the groups. Therefore, the mechanism by which high levels of GAA alter splenic glutamine is unclear.

Each organ has a unique metabolic function, that is, the digestive and immune functions of the liver, the waste excretion by the kidney, the maintenance of blood circulation by the heart, and the immune function of the spleen. The metabolism of these organs is highly active. These organs produce or store compounds besides using other nutrients [49]. Torell et al. [49] reported that the pancreas had the smallest contribution to the level of plasma metabolites, which was similar to the results of this study. That is, the correlation of the liver, kidney, spleen, and heart to the plasma amino acid profile was higher than that of the pancreas to the plasma amino acid profile. Significant correlations existed between amino acid profiles, reflecting shared biochemical pathways [50]. However, the mechanism by which GAA changes the tissue amino acid profile is still unclear. If the isotope tracking method is used to track the catabolism of GAA, the mechanism of GAA changing amino acid distribution can be better understood.

## Conclusion

It was concluded that increasing dietary GAA supplementation had a significant effect on the tissue distribution of specific amino acids. GAA supplementation altered pig plasma and tissue free amino acid profiles and the contents of related metabolites content in pigs in a tissue-dependent manner. The main findings observed in animals supplemented with a GAA diet included the reductions of many amino acid concentrations in plasma and tissues. However, the present study still had some limitations. First, the amino acid profile of the tissues studied did not represent all the tissues of the body, especially the skeletal muscle with the largest amount of tissue, the brain with high energy metabolism, and the tissues and organs of the digestive system (stomach and gut). Second, the amino acid profile was not as good as amino acid metabolomics, which was more suitable to explain the changes in amino acid metabolism in tissues. In addition, a detailed understanding of the mechanism by which GAA affects protein metabolism in finishing pigs is needed. Further studies should be conducted to understand the mechanism of GAA changing amino acid distribution. Also, we should explore the changes in amino acid transporters, amino acid synthase, and GAA anabolism-related enzymes involved.

## Materials and methods

### Experimental design, animals, and diets

Guanidine acetic acid (GAA) was provided by Guangdong Newland Feed Science Technology Co., Ltd. (Guangzhou, China). A total of 72 140-day old (body weight 86.59 ± 1.16 kg) crossbred pigs (Duroc × Landrace × Large White) were used. The pigs were randomly assigned to one of four dietary treatments (GAA0, GAA500, GAA1000, and GAA1500) in a completely randomized design, which were fed the basal diets supplemented with 0, 500, 1000, or 1500 mg/kg GAA, respectively. Each treatment comprised 6 replicate pens, with 3 barrows in each. The experimental period lasted 42 days. As shown in Table 10, all diets were formulated to meet or excess the nutrient requirements suggested by NRC 2012 (75–100 kg growing pig). All pigs had ad libitum access to feed and water during the experimental period.

**Table 10:** Composition and nutrient content of the basal diet (%, as-fed basis)

Ingredients	Content	Nutrient composition ^b^	Content
Corn	71.00	Metabolizable energy, MJ/kg	14.27
Soybean meal	16.98	Net energy, MJ/kg	10.40
Wheat bran	6.00	Crude protein	14.53
Soybean oil	2.50	Calcium	0.61
Salt	0.30	Total phosphorus	0.50
CaHPO_4_	0.95	Available phosphorus	0.23
Limestone	0.75	Standardized ileal digestible amino acids	
L-Lysine	0.38	Lysine	0.88
DL-Methionine	0.06	Methionine + Cystine	0.47
L-Threonine	0.06	Threonine	0.49
L-Tryptophan	0.02	Tryptophan	0.15
Premix ^a^	1.00		
Total	100.00		

^a^Premix is provided for each kg of diet: Vitamin A 7750 IU, VitaminD_3_ 1750 IU, Vitamin E 19 IU, Vitamin K 3 mg, Vitamin B_12_ 25 μg, Vitamin B_1_ 1.9 mg, Vitamin B_2_ 6 mg, nicotinic acid 25 mg, D-pantothenic acid 9 mg, folic acid 0.6 mg, Vitamin B_6_ 5 mg, biotin 0.05 mg, FeSO_4_·H_2_O 72 mg, CuSO_4_·5H_2_O 10 mg, MnSO_4_·H_2_O 42 mg, ZnSO_4_·H_2_O 72 mg, CaI_2_O_6_ 0.42 mg, Na_2_SeO_3_ 0.29 mg

^b^Calculated value. The values are expressed as percentage (%), except for digestible energy and net energy

### Growth performance

The pigs were weighed individually on days 1, 21, and 42, and the feed consumption per pen was measured daily. The average daily feed intake, average daily gain, and feed/gain were calculated.

### Slaughter procedure, sample collection, and processing

After fasting for approximately 12 h, the pigs closest to the average weight of treatments were slaughtered. Blood collection from pig ear veins using heparin sodium evacuated tubes (YL012, Yuli, Jiangsu, China). Blood was centrifuged at 1800 × *g* for 10 min at 4℃, and plasma was immediately placed in liquid nitrogen after collection, and then stored at  − 80 ℃. The pigs were stunned by electro-anesthesia and killed by throat slitting, according to current slaughterhouse practices. The heart, liver, spleen, pancreas, and kidneys were removed immediately after slaughter, weighed, sampled, and frozen in liquid nitrogen quickly, and then stored at − 80 ℃. The remaining tissue samples were weighed separately, cut into small pieces, and then freeze-dried (ALPHA 2-4 LSC, Martin Christ GmbH, Osterode am Harz, Germany). The freeze-dried tissue samples were maintained at − 80 ℃ for crude protein and amino acid analysis.

### Crude protein analysis

The crude protein content of tissues was analyzed by the Kjeldahl method using an 8400 Automatic Nitrogen Analyzer (FOSS, Hillerod, Denmark).

### Free amino acid analysis

Plasma (0.4 mL) and 10% sulfosalicylic acid (1.2 mL) were vortexed for 15 min and then centrifuged at 12,000 × *g* for 15 min at 4 °C to remove proteins. Freeze-dried tissue samples (0.2 g) and 10% sulfosalicylic acid (1.5 mL) were homogenized for 15 min and then centrifuged at 12,000 × *g* for 15 min at 4 ℃. The supernatant was filtered with a 0.22-μm filter, and then measured using an amino acid analyzer (L-8900, Hitachi Ltd., Tokyo, Japan). The amino acid concentrations in plasma were expressed as nmol/mL and those in tissues were expressed as g/kg dry tissue. Essential amino acids (EAA) included lysine, methionine, tryptophan, threonine, arginine, histidine, leucine, isoleucine, phenylalanine and valine, nonessential amino acids (NEAA) = total amino acids (TAA)–EAA, BCAA included leucine, isoleucine, and valine.

### Statistical analysis

All data were computed using the one-way ANOVA in SPSS 25.0 (SPSS Inc., Chicago, IL, USA). Differences among treatments were separated by Tukey's multiple range test. Linear and quadratic effects were evaluated within increasing GAA treatments with orthogonal-polynomial contrasts [51]. Spearman coefficient was used to analyze the relationship between plasma and tissue free amino acids. Figures were prepared using Graphpad Prism 8.0 (GraphPad Software, lnc., La Jolla, CA). Results are expressed as means and standard error of the mean (SEM), and a significance level of 0.05 was used.

## Data Availability

The datasets supporting the conclusions of this article are included within the article.

## References

[1] Ostojic SM (2016). Guanidinoacetic acid as a performance-enhancing agent. Amino Acids.

[2] Dao HT, Sharma NK, Bradbury EJ, Swick RA (2021). Response of laying hens to l-arginine, l-citrulline and guanidinoacetic acid supplementation in reduced protein diet. Anim Nutr.

[3] Nasirikhah A, Zhandi M, Shakeri M, Sadeghi M, Ansari M, Deldar H (2019). Dietary guanidinoacetic acid modulates testicular histology and expression of c-Kit and STRA8 genes in roosters. Theriogenology.

[4] Ostojic SM (2017). Tackling guanidinoacetic acid for advanced cellular bioenergetics. Nutrition.

[5] Poaa EFSA (2016). Safety and efficacy of guanidinoacetic acid for chickens for fattening, breeder hens and roosters, and pigs. EFSA J.

[6] U. S. Food and Drug Administration [FDA]. Food additives permitted in feed and drinking water of animals, guanidinoacetic acid. US FDA. 2016. https://www.federalregister.gov/documents/2016/11/30/2016-28754/food-additi-ves-permitted-in-feed-and-drinking-water-of-animals-guanidinoacetic-acid

[7] MOA Ministry of Agriculture (PRC). Bulletin of the ministry of agriculture of the People's Republic of China. MOA of PRC. 2014. http://jiuban.moa.gov.cn/zwllm/tzgg/gg/201411/t20141115_4209901.htm

[8] Majdeddin M, Golian A, Kermanshahi H, Michiels J, De Smet S (2019). Effects of methionine and guanidinoacetic acid supplementation on performance and energy metabolites in breast muscle of male broiler chickens fed corn-soybean diets. Br Poult Sci.

[9] Jayaraman B, La KV, La H, Doan V, Carpena EM, Rademacher M (2018). Supplementation of guanidinoacetic acid to pig diets: effects on performance, carcass characteristics, and meat quality. J Anim Sci.

[10] He DT, Gai XR, Yang LB, Li JT, Lai WQ, Sun XL (2018). Effects of guanidinoacetic acid on growth performance, creatine and energy metabolism, and carcass characteristics in growing-finishing pigs. J Anim Sci.

[11] Zhu Z, Gu C, Hu S, Li B, Zeng X, Yin J (2020). Dietary guanidinoacetic acid supplementation improved carcass characteristics, meat quality and muscle fibre traits in growing–finishing gilts. J Anim Physiol Anim Nutr.

[12] de Souza C, Eyng C, Viott AM, de Avila AS, Pacheco WJ, Junior NR (2021). Effect of dietary guanidinoacetic acid or nucleotides supplementation on growth performances, carcass traits, meat quality and occurrence of myopathies in broilers. Livest Sci.

[13] Yang L, Wu P, Feng L, Jiang W, Liu Y, Kuang S (2021). Guanidinoacetic acid supplementation totally based on vegetable meal diet improved the growth performance, muscle flavor components and sensory characteristics of on-growing grass carp (Ctenopharygodon idella). Aquaculture.

[14] Ahmadipour B, Naeini SZ, Sharifi M, Khajali F (2018). Growth performance and right ventricular hypertrophy responses of broiler chickens to guanidinoacetic acid supplementation under hypobaric hypoxia. J Poult Sci.

[15] Majdeddin M, Braun U, Lemme A, Golian A, Kermanshahi H, De Smet S (2020). Guanidinoacetic acid supplementation improves feed conversion in broilers subjected to heat stress associated with muscle creatine loading and arginine sparing. Poult Sci.

[16] Ale Saheb Fosoul SS, Azarfar A, Gheisari A, Khosravinia H (2019). Performance and physiological responses of broiler chickens to supplemental guanidinoacetic acid in arginine-deficient diets. Brit Poul Sci.

[17] Zarghi H, Golian A, Tabatabaei YF (2020). Effect of dietary sulphur amino acid levels and guanidinoacetic acid supplementation on performance, carcase yield and energetic molecular metabolites in broiler chickens fed wheat-soy diets. Ital J Anim Sci.

[18] Dilger RN, Bryant-Angeloni K, Payne RL, Lemme A, Parsons CM (2013). Dietary guanidino acetic acid is an efficacious replacement for arginine for young chicks. Poult Sci.

[19] Zhang L, Li JL, Wang XF, Zhu XD, Gao F, Zhou GH (2019). Attenuating effects of guanidinoacetic acid on preslaughter transport-induced muscle energy expenditure and rapid glycolysis of broilers. Poult Sci.

[20] Stajer V, Trivic T, Drid P, Vranes M, Ostojic SM (2016). A single session of exhaustive exercise markedly decreases circulating levels of guanidinoacetic acid in healthy men and women. Appl Physiol Nutr Metab.

[21] Wu G (2020). Important roles of dietary taurine, creatine, carnosine, anserine and 4-hydroxyproline in human nutrition and health. Amino Acids.

[22] Suzuki A, Iwata J (2021). Amino acid metabolism and autophagy in skeletal development and homeostasis. Bone.

[23] Fernández-Fígares I, Nieto R, Aguilera JF, Lachica M (2014). Changes in tissue free amino acid pools in growing chickens fed thermally treated vetch diets. J Anim Physiol Anim Nutr.

[24] Conde-Aguilera JA, Le Floc HN, Le Huërou-Luron I, Mercier Y, Tesseraud S, Lefaucheur L (2016). Splanchnic tissues respond differently when piglets are offered a diet 30 % deficient in total sulfur amino acid for 10 days. Eur J Nutr.

[25] Wang LS, Shi BM, Shan AS, Zhang YY (2012). Effects of guanidinoacetic acid on growth performance, meat quality and antioxidation in growing-finishing pigs. J Anim Vet Adv.

[26] McBreairty LE, Robinson JL, Furlong KR, Brunton JA, Bertolo RF (2015). Guanidinoacetate is more effective than creatine at enhancing tissue creatine stores while consequently limiting methionine availability in Yucatan miniature pigs. PLoS ONE.

[27] Li J, Zhang L, Fu Y, Li Y, Jiang Y, Zhou G (2018). Creatine monohydrate and guanidinoacetic acid supplementation affects the growth performance, meat quality, and creatine metabolism of finishing pigs. J Agric Food Chem.

[28] Duranton F, Lundin U, Gayrard N, Mischak H, Aparicio M, Mourad G (2014). Plasma and urinary amino acid metabolomic profiling in patients with different levels of kidney function. Clin J Am Soc Nephrol.

[29] Li Q, Yan Q, Zhou C, Tang S, Han X, Tan Z (2021). Effects of dietary zinc-methionine supplementation during pregnancy on the whole-genome methylation and related gene expression in the liver and spleen of growing goats: a short communication. Biol Trace Elem Res.

[30] Cao YC, Yang XJ, Guo L, Zheng C, Wang DD, Cai CJ (2018). Effects of dietary leucine and phenylalanine on pancreas development, enzyme activity, and relative gene expression in milk-fed Holstein dairy calves. J Dairy Sci.

[31] Elefson SK, Lu N, Chevalier T, Dierking S, Wang D, Monegue HJ (2021). Assessment of visceral organ growth in pigs from birth through 150 kg. J Anim Sci.

[32] Wang W, Wu Z, Dai Z, Yang Y, Wang J, Wu G (2013). Glycine metabolism in animals and humans: implications for nutrition and health. Amino Acids.

[33] Siegert W, Rodehutscord M (2019). The relevance of glycine and serine in poultry nutrition: a review. Br Poultry Sci.

[34] Alves A, Bassot A, Bulteau A (2019). Glycine metabolism and its alterations in obesity and metabolic diseases. Nutrients.

[35] Ardalan M, Batista ED, Titgemeyer EC (2020). Effect of post-ruminal guanidinoacetic acid supplementation on creatine synthesis and plasma homocysteine concentrations in cattle. J Anim Sci.

[36] DeGroot AA, Braun U, Dilger RN (2018). Efficacy of guanidinoacetic acid on growth and muscle energy metabolism in broiler chicks receiving arginine-deficient diets. Poult Sci.

[37] Liu C, Wang C, Zhang J, Liu Q, Guo G, Huo WJ (2021). Guanidinoacetic acid and betaine supplementation have positive effects on growth performance, nutrient digestion and rumen fermentation in Angus bulls. Anim Feed Sci Tech.

[38] He D, Yang L, Li J, Dong B, Lai W, Zhang L (2019). Effects of guanidinoacetic acid on growth performance, creatine metabolism and plasma amino acid profile in broilers. J Anim Physiol Anim Nutr.

[39] Ostojic SM (2015). Advanced physiological roles of guanidinoacetic acid. Eur J Nutr.

[40] Ma B, Zhang L, Li J, Xing T, Jiang Y, Gao F (2021). Heat stress alters muscle protein and amino acid metabolism and accelerates liver gluconeogenesis for energy supply in broilers. Poult Sci.

[41] Ostojic SM, Niess B, Stojanovic M, Obrenovic M (2013). Creatine metabolism and safety profiles after six-week oral guanidinoacetic acid administration in healthy humans. Int J Med Sci.

[42] Du Y, Xu B, Deng X, Wu X, Li Y, Wang S (2019). Predictive metabolic signatures for the occurrence and development of diabetic nephropathy and the intervention of Ginkgo biloba leaves extract based on gas or liquid chromatography with mass spectrometry. J Pharmaceut Biomed.

[43] Neis EPJG, Sabrkhany S, Hundscheid I, Schellekens D, Lenaerts K, Olde Damink SW (2017). Human splanchnic amino-acid metabolism. Amino Acids.

[44] Jang C, Hui S, Zeng X, Cowan AJ, Wang L, Chen L (2019). Metabolite exchange between mammalian organs quantified in pigs. Cell Metab.

[45] Shi C, Wang L, Zhou K, Shao M, Lu Y, Wu T (2020). Targeted metabolomics identifies differential serum and liver amino acids biomarkers in rats with alcoholic liver disease. J Nutr Sci Vitaminol.

[46] Feng L, Liu X, Cao F, Wang L, Chen Y, Pan R (2016). Anti-stress effects of ginseng total saponins on hindlimb-unloaded rats assessed by a metabolomics study. J Ethnopharmacol.

[47] Anton Dib Saleh M, Sousa L, dos Santos D, Berto A, Amorim AB, Tse MLP, Costa VE (2019). IRMS as a tool to obtain the carbon turnover (δ13C) in organs of weaned piglets fed glutamic acid and nucleotides. J Anim Physiol Anim Nutr.

[48] Wang Y, Han G, Pham CV, Koyanagi K, Song Y, Sudo R (2019). An acute increase in water temperature can increase free amino acid concentrations in the blood, brain, liver, and muscle in goldfish (Carassius auratus). Fish Physiol Biochem.

[49] Torell F, Bennett K, Cereghini S, Rännar S, Lundstedt-Enkel K, Moritz T (2015). Multi-organ contribution to the metabolic plasma profile using hierarchical modelling. PLoS ONE.

[50] Ponsuksili S, Trakooljul N, Hadlich F, Methling K, Lalk M, Murani E (2019). Genetic regulation of liver metabolites and transcripts linking to biochemical-clinical parameters. Front Genet.

[51] Maxwell SE, Delaney HD, Kelley K (2017). Designing experiments and analyzing data: a model comparison perspective.

